# Physiotherapy-integrated yoga and mindfulness plus home exercise versus home exercise alone for individuals with fibromyalgia syndrome (PhYoMind): study protocol of a randomised controlled clinical trial

**DOI:** 10.1136/bmjopen-2026-120248

**Published:** 2026-07-06

**Authors:** Hazal Sarak Kucukosmanoglu, Aydan Aytar, Mirela-Ioana Bilc, Dennis Anheyer, Alexandra N V Kieninger, Anita Alaze, Holger Cramer

**Affiliations:** 1 Institute of General Practice and Interprofessional Care, University Hospital Tübingen, Tübingen, Germany; 2 Robert Bosch Center for Integrative Medicine and Health, Bosch Health Campus, Stuttgart, Germany; 3 Gülhane Faculty of Physiotherapy and Rehabilitation, University of Health Sciences, Ankara, Turkey; 4 Chair of Research Methodology and Statistics in Psychology, Department of Psychology and Psychotherapy, University Witten Herdecke, Witten, Germany

**Keywords:** Randomized Controlled Trial, Fibromyalgia, Mindfulness, Chronic Pain, REHABILITATION MEDICINE, Physical Therapy Modalities

## Abstract

**Background:**

Fibromyalgia syndrome (FMS) is a chronic condition characterised by the presence of complex multiple symptoms, often accompanied by psychiatric comorbidities and sleep problems. The wide spectrum of symptoms and comorbid problems complicates treatment and management of the ensuing disability and symptoms. Physiotherapy approaches and mind-body practices including yoga and mindfulness are among the non-pharmacological treatment methods commonly used in FMS treatment. While there is initial evidence for these interventions, definitive conclusions about their effectiveness are still lacking and research on their combined effectiveness is limited. Therefore, this study aims to evaluate the effectiveness of a multimodal integrative protocol, the PhYoMind (PYM) intervention, which combines specific physical therapy modalities with yoga and mindfulness practices on the overall impact of fibromyalgia and functional impairment.

**Methods and analysis:**

This monocentric study uses a parallel-group (1:1) randomised controlled design, in which the outcome assessor and statistician are blinded to group allocation. Individuals with a clinical diagnosis of FMS (n=40) will be included in the study (Cohen’s f=0.675; α=0.05; power=0.80). Participants will be randomised to receive either PYM intervention in addition to Home Exercise (HE) programme or HE programme alone. The intervention period for both groups will last 8 weeks; PYM sessions will be held twice a week for 75 min each. The primary outcome is the broad disease impact and functional impairments measured by the total score of the Fibromyalgia Impact Questionnaire. Secondary outcomes include central sensitivity, as assessed by the Central Sensitisation Index; autonomic nervous system function, as measured objectively by heart rate variability; pain perception (current, average, worst), as assessed by the Visual Analogue Scale; fatigue, as assessed by the Multidimensional Fatigue Inventory; stress, as assessed by the Perceived Stress Scale; and sleep quality as assessed by the Pittsburgh Sleep Quality Index. Adherence and adverse events will be assessed for both interventions. Repeated measures ANOVA will be used to analyse the effect of time, intervention group and their interaction on primary and secondary outcomes under an intention-to-treat principle.

**Ethics and dissemination:**

Ethics approval was granted from the Ethics Committee at the Medical Faculty of Eberhard Karls Tuebingen University and at the University Hospital of Tuebingen (260/2025BO2) on 30 April 2025. The trial will be conducted in accordance with the updated principles of the Declaration of Helsinki. The study’s findings will be disseminated and documented in a peer-reviewed publication, adhering to the Consolidated Standards of Reporting Trials guidelines. After completion of the study and publication of the results, participants who are interested will be offered a brief summary of the aggregated study findings.

**Trial registration number:**

NCT07145788.

**STRENGTHS AND LIMITATIONS OF THIS STUDY**

- This study addresses a crucial evidence gap by evaluating an innovative, multimodal, integrative intervention that combines specific physiotherapy techniques with yoga and mindfulness in patients with fibromyalgia syndrome (PhYoMind), with the aim of addressing various relevant domains of the condition.
- By comparing PhYoMind intervention plus home exercise with home exercise alone, the study may help clarify whether the combined intervention provides incremental benefits beyond exercise alone, thereby informing future research on complementary, non-pharmacological interventions for fibromyalgia syndrome.
- The follow-up period is limited to 8 weeks in this study, which limits the ability to draw conclusions regarding the long-term sustainability of the treatment effects.

## Introduction

 Fibromyalgia syndrome (FMS) is a chronic condition characterised by widespread and persistent non-inflammatory musculoskeletal pain, morning stiffness, fatigue, sleep disturbances, depression, anxiety, cognitive and emotional dysfunctions, and abnormalities in the central and autonomic nervous system.^1^,^4^ Although the etiopathogenesis of FMS has not been fully elucidated, genetic, neurological, psychological, sleep-related and immunological factors have been proposed as contributing factors.^5^ The worldwide prevalence of FMS ranges between 2 and 8%, depending on the diagnostic criteria used, and FMS is more common in women than in men.^6 7^ Abnormal functional neuroimaging of individuals with FMS shows that the central nervous system (CNS) is affected.^8^ Dysfunction in pain processing areas such as the insula, increased concentrations of pronociceptive neurotransmitters (eg, substance P and glutamate) in cerebrospinal fluid, dopamine dysregulation and decreased serotonin and norepinephrine in descending anti-nociceptive pathways all support CNS involvement.^8^,^10^ However, some symptoms of FMS cannot be explained solely by CNS abnormalities.^11^ They are also linked to the autonomic nervous system (ANS), including the hypothalamo-pituitary-adrenal (HPA) axis^11 12^ dysfunction. Multiple comorbidities and accompanying symptoms, in particular chronic fatigue, depression, anxiety, sleep and cognitive dysfunctions,^4 13 14^ can be explained by ANS dysfunction,^15^ suggesting that altered allostatic regulation contributes to FMS beyond CNS abnormalities.^16^

Mind-body interventions (MBIs) such as mindfulness and yoga appear to modulate pain by altering attentional deployment and cognitive control mechanisms.^17 18^ They are thought to improve cognitive flexibility by enabling the person to see their emotional reactions from an objective perspective without judgement, which could alleviate FMS-related symptoms.^19 20^ MBIs can also enhance the quality of life by improving the ability to cope with pain-related psychological problems such as stress, anxiety and depression.^21^ Furthermore, allostasis has been reported as a mechanism underlying the effectiveness of MBIs in chronic pain disorders similar to FMS,^16^ where changes in HPA-axis activity and ANS function may lead to structural brain adaptations that facilitate coping with internal/external demands.^22^ Nevertheless, recent systematic reviews have found a paucity of high-quality evidence supporting MBIs, including yoga- and mindfulness-based approaches, in the management of FMS symptoms.^23 24^ Accordingly, more randomised controlled trials have been recommended to investigate the additive or synergistic effects of different exercise modalities or techniques in combination with MBIs.^25^ In addition, the German S3 guideline for FMS strongly recommends the incorporation of meditative movement therapies, including yoga, emphasising multimodal treatment programmes.^26^

In line with the National Center for Complementary and Integrative Health’s conceptualisation of integrative approaches as a coordinated multimodal care strategy,^27^ which integrates conventional and complementary methodologies, the current study proposes a multimodal integrative approach. It has been developed specifically for this trial, integrating physiotherapy techniques into a structured yoga sequence and combining them with mindfulness principles. Neuromusculoskeletal function, pain-related biopsychological mechanisms, autonomic regulation and stress-related mechanisms are targeted simultaneously. The aim is to combine physiotherapy approaches targeting pain modulation and physical function with yoga- and mindfulness-based practices that may support stress regulation, sleep and fatigue, thereby providing an integrated approach to the multidomain symptom profile of fibromyalgia. This programme, termed PhYoMind (PYM), has been developed by combining various components whose effectiveness in addressing the different mechanisms underlying specific FMS symptoms has been suggested in a number of studies. PYM integrates specific physiotherapy techniques including Proprioceptive Neuromuscular Facilitation (PNF), Nerve Gliding Exercises (NGE), Mobility Exercises (ME) and Post-Isometric Relaxation (PIR) with yoga and mindfulness. Evidence from physiotherapy research suggests that several approaches incorporated in our intervention may help prevent declines in physical function in FMS and improve symptoms such as pain, limited range of motion, balance impairments, morning stiffness and strength.^28^,^30^ For example, PNF may increase short-term range of motion via autogenic/reciprocal inhibition; while long-term mobility gains may reflect increased tolerance through pain modulation.^31^ NGE has been shown to improve pain, functional status and fatigue in FMS, possibly through inhibition of central sensitisation, improved nerve blood flow and reduced mechanical and inflammatory stress around nerves.^28^ Another randomised controlled trial (RCT) showed that both PIR and myofascial release improved cervical and low back pain, mobility and quality of life in women with FMS.^32^ Apart from the benefits these various techniques offer on their own or as part of physiotherapy programmes, they are very well suited to yoga-based practices because they can be applied gently, gradually and tailored to the individual’s tolerance under in-person instruction and supervision. This is particularly important for FMS patients, who often have limited tolerance for high-intensity exercise.^33^ Given the complex and multifactorial nature of FMS symptoms, the structure of the PYM intervention, which addresses numerous different mechanisms, and the integration of these under a single intervention framework, along with its comprehensive structure, makes it a highly innovative intervention.

There are several FMS major guidelines that can be applied in the German context; these recommend various therapeutic approaches for the different treatment of FMS, including exercise.^26 34 35^ One of these guidelines is the European Alliance of Associations for Rheumatology (EULAR) guideline, which applies to the European context and recommends different non-pharmacological treatments, such as exercise, meditative movement and mind-body therapy for FMS. In this guideline, exercise is the only non-pharmacological treatment for FMS that is strongly recommended (strong for 100% agreement) based on high-quality evidence.^34^ The EULAR explicitly highlights aerobic and strength training as effective types of exercise for FMS.^34^ In line with this, the German interdisciplinary S3 guideline on FMS emphasises the importance of exercise (aerobic, strength and flexibility) as an intervention supported by high level-evidence and a strong recommendation.^26^ Moreover, the American College of Sports Medicine (ACSM) provides guidance on exercise dosing and prescription, which has been used to evaluate the adherence of exercise interventions to recommended dosage parameters in FMS.^36 37^ To provide a more rigorous comparison in this study, the Home Exercise (HE) programme comprises all these evidence-based recommendations: aerobic, strength and stretching exercises with the recommended dosing. Both groups receive the HE programme, while the intervention group additionally participates in PYM sessions, allowing evaluation of the added benefit of PYM beyond HEs alone.

PYM intervention stands out for its unique approach to addressing FMS symptoms; this programme, in which the techniques used in physiotherapy, yoga and mindfulness components are presented as a cohesive whole rather than as discrete modules, represents a distinctive within an integrated, replicable programme. Beyond this, the fact that the intervention group carries out both PYM and HEs has the potential to yield greater synergistic benefits for the intervention group. To the best of our knowledge, no existing research has examined the effect of integrating certain physiotherapy techniques with yoga and mindfulness in FMS patients, as proposed in this study. Therefore, the primary objective of this trial is to evaluate whether an 8-week PYM intervention in addition to HEs is superior to HEs alone in improving fibromyalgia symptom severity and functional impact after the intervention in adults with FMS. Secondary objectives are to compare the effects of the PYM and HE combination on central sensitivity, autonomic nervous system function, pain perception, perceived stress, fatigue and sleep quality with those of HEs alone. Furthermore, the study aims to monitor adverse events to assess the safety of both interventions and evaluate adherence and attendance as feasibility outcomes.

## Methods and design

This protocol for the randomised controlled clinical trial has been reported in accordance with the most recent 2025 revision of the SPIRIT statement.^38^ The study is currently being conducted, and participant recruitment has commenced.

### Study design

This monocentric study uses a superiority randomised controlled design with parallel allocation, comparing two groups in a 1:1 ratio with both the outcome assessors and the statisticians performing the data analysis blinded to group allocation.

### Study settings and recruitment

Potential participants will be recruited from the greater Stuttgart area through external channels (eg, social media campaigns, flyers, outpatient clinics, FMS self-help groups in Stuttgart, community advertising). Advertising activities will be supported by the communication department of the research centre. In addition, the study will be presented in person to local FMS self-help groups by members of the study team. The trial will be conducted at the Bosch Health Campus, Stuttgart, Germany and interventions are planned to be carried out between March and July 2026.

### Eligibility criteria

Interested individuals who have become aware of the study through the aforementioned channels will contact the study team by phone or e-mail. A telephone pre-screening procedure is conducted to determine the likely presence of FMS based on a recognised diagnosis and current symptoms. If the pre-screening yields positive results, interested patients will be invited to the Bosch Health Campus, where eligibility will then be assessed by detailed clinical examination, including confirmation of the fibromyalgia diagnosis^39 40^ and verification of all inclusion and exclusion criteria by the study physician.

The following inclusion and exclusion criteria are defined for participation:

Inclusion criteria:

- Age ≥18 years.
- Clinically diagnosed FMS (it will be confirmed using the 2016 American College of Rheumatology (ACR) diagnostic criteria,^40^ which incorporate the severity of symptoms and require the presence of widespread pain. These criteria are the most current, widely used in both clinical and research contexts).

Exclusion criteria:

- Other serious illnesses causing severe pain and complicating participation in the study.
- Receiving another physiotherapy treatment concurrently with this study.
- Regular practice of yoga and/or mindfulness exercises prior to the study.
- History of acute myocardial infarction or heart failure and resulting cardiac capacity limitations.
- Uncontrolled hypertension (resting systolic/diastolic blood pressure ≥160/100 mm Hg).
- Current treatment with medications known to significantly affect heart rate variability (HRV) (eg, beta-blockers or antiarrhythmic drugs).
- Pregnancy or planned pregnancy during the study period.
- Severe psychiatric disorders or insufficient cognitive abilities resulting in inability to participate in the study.
- Participants must either be on a stable treatment for FMS or not receiving any treatment, with no intention to alter their treatment plan during the study period. (Any unintended changes in fibromyalgia-related medications or other treatments during the study will be recorded and documented).

Eligible patients will be included in the study and will sign the written informed consent form prior to any study procedures (see onlinesupplemental files 1  2: Patient Consent Form). Baseline measurements will be conducted on the same day. In addition, on the same appointment, all included participants will have a face-to-face session with study personnel and will be taught the HEs based on a HE brochure and will be asked to follow the prescribed HEs once the study intervention begins via email. Participants in both groups may continue their usual medical care, including ongoing FMS medication at a stable dose where possible, with no study mandated changes as well as routine primary care. However, the initiation of new treatments that could directly influence the trial’s outcomes, such as additional physiotherapy, structured exercise programmes or yoga- and mindfulness-based interventions, or any medication changes targeting pain or fibromyalgia, should be avoided for the remainder of the study. Such cases will be documented by the study team.

### Intervention and comparator

PYM intervention will be carried out in a dedicated exercise room at Bosch Health Campus, which is equipped with the necessary materials for the sessions. PYM sessions will be conducted by the study physician (ANVK), who is an orthopaedic specialist and experienced yoga instructor with 840-hour yoga teacher training. One of the authors (HSK), a physiotherapist and also a certified yoga instructor with 420-Hour yoga teacher training, will provide detailed instruction on the PYM protocol to the study physician prior to study start, and together they will define the structure and content of intervention sessions to ensure consistency, fidelity to the protocol and safe delivery of the intervention.

#### Intervention group: supervised PYM sessions+home exercise

Intervention group participants will receive both the supervised PYM intervention and an unsupervised home-exercise programme (see detailed description: Active Control Group). Participants in the intervention group will be divided into two subgroups of up to 10 participants. Each subgroup will attend 16 supervised PYM sessions over 8 weeks (two times per week, 75 min per session) and complete 16 HE sessions in the same period (two times per week, 60 min per session). This subgroup division is intended to facilitate appropriate modification of the practice to participants’ individual limitations and to allow closer observation during the PYM sessions. In addition, the intervention will be delivered in successive cohorts, with short gaps between cohorts to accommodate assessment scheduling. The sessions will follow the structure outlined in table 1, and verbal and tactile adjustments will be made if necessary.

**Table 1:** PhYoMind intervention structural framework

PhYoMind intervention			
At the beginning of each session, setting an intention and inviting practice accordingly			
1.	Body tapping, full body shaking, dynamic warm-up sequences or easy sun salutations		10 min
2.	Standing poses	Physiotherapy techniques will be used in yoga poses: Self-proprioceptive neuromuscular facilitation techniques*, self-nerve gliding techniques*†**, self-mobility exercises**‡*	40 min
3.	Back bend-focused poses		
4.	Forward bend-focused poses		
5.	Three block poses (hip openers/strengtheners, core strengtheners, spinal rotation or balance-oriented poses)		
6.	Inversion pose (legs up the wall pose)		2 min
7.	Breathing practices (alternate/left nostril breathing and humming bee breathing)		8 min
8.	Mindfulness meditation		5 min
9.	Shavasana pose with post isometric relaxation§		10 min

* Contract-relax technique, contract-relax antagonist contract technique.

† Median nerve, ulnar nerve, radian nerve, sciatic nerve, femoral nerve gliding.

‡ Shoulder, spine and hip mobility exercises.

§ Sequential submaximal isometric contractions of major muscle groups from feet to head, followed by one whole body squeeze-release, then resting in Shavasana.

As seen in table 1, body tapping will be performed at the beginning of the session to warm up the body. Body tapping involves meridian pathways referring to acupoints, specific and anatomically standardised locations on the body in Traditional Chinese Medicine.^41^ In accordance with the WHO’s standard nomenclature, these acupoint-based meridian pathways incorporate various organ meridians, including the lung, large intestine, heart, kidney and liver meridians, among others.^42^ The body tapping procedure is initiated in an upright position with feet shoulder-width apart and knees slightly bent. Gentle bouncing movements are performed while tapping and scanning all these pathways in a sequence using both hands, starting from the head and progressing to the legs, similar to the intervention procedure described by Neimeyer.^43^ The tapping sequence progresses through the top of the skull, face, chest, shoulders, arms (forearm to palm and back for each side), upper and lower abdomen, front and back, outer and inner thighs, concluding with gently full body shaking movement. Dynamic warm-up sequence or sun salutations (Surya Namaskar), modified for ease of performance, will then be performed, just as in a regular yoga session, to warm up the body. In order to ensure that participants can comfortably perform the sun salutation movements, these movements will be performed as a simplified sequence. For instance, rolling the spine up and down to move from mountain pose into a forward bend, or flowing smoothly from easy cobra pose into elbow plank pose. Then, yoga poses will be implemented in the order of a typical hatha yoga planning for 40 min. Each session will focus on different parts of the body, and yoga poses will be selected according to these focused areas, as well as the spiritual and philosophical intent of that day’s session and will be planned using the Tummee Yoga Sequencing Platform.^44^ The participants’ needs and their physical abilities will also be taken into consideration. For instance, the first session will focus on the hip region and lower extremities, the second session on the upper body and upper extremities, the third session on the core and anterior myofascial line, and the fourth session on the back and posterior myofascial line. These focus areas will then be repeated in the similar sequence until the final week. An example of a PYM session is described in detail in online supplemental file 3 (Example PhYoMind session).

#### Active control group: home-exercise

The control group will receive a simplified, guideline-based home-exercise (HE) programme (see online supplemental file 4: home-exercise brochure). The HE programme is prescribed taking into account the key FITT parameters (Frequency, Intensity, Time and Type) consistent with the core exercise-related recommendations of EULAR, ACSM and the German interdisciplinary guideline for FMS.^26 34 36^ Once the intervention period begins, participants will perform the HE programme at home twice per week for 8 weeks (16 sessions in total), with each session lasting approximately 60 min. Each session will comprise three phases. First, there will be 30 min of walking at a pace that is moderately fast and tolerable to the individual. Walking intensity will be prescribed using the Talk Test, in line with guidance for monitoring aerobic exercise intensity.^45^ Second, there will be 15 min of strength exercises (2–3 sets of 8–12 repetitions with body weight and/or tolerable external load) targeting the back extensor muscles, core stabilisation muscles, shoulder-girdle muscles of the upper extremity and hip and knee muscles of the lower extremity; no structured progression will be implemented. Thirdly, there will be 15 min of stretching exercises for the neck muscles, pectoralis major/minor, erector spinae, hamstrings and quadriceps femoris (one to two repetitions of 30 s each).

In order to enhance safety and feasibility, participants will be instructed in symptom-contingent pacing and pain flare. If pain markedly increased or a flare occurred, participants will be advised to reduce exercise dose (by decreasing range of motion, sets/repetitions, walking pace/duration or by selecting easier variations). They will also be advised to temporarily pause if necessary and to resume at the previously tolerated level once symptoms have stabilised.

In addition, as an incentive to enhance adherence in the control group, participants will be offered 6 PYM intervention sessions after completion of all trial measurements.

### Adverse events and adherence of interventions

Adverse events will be measured through predefined items in exercise diaries for both groups. Individuals in both intervention and control groups will receive the exercise diary every 2 weeks via an emailed REDCap link (see onlinesupplemental files 5  6: Intervention/Control Group Exercise Diary). They will complete it retrospectively for the preceding four exercise sessions and submit it electronically through REDCap. If no email address is available, the diary will be sent/returned by post and responses will be entered into REDCap by the study team. Subsequently, all intervention-related events will be reviewed by the study team and classified according to predefined criteria including intervention context (supervised PYM vs HE), event type (physical, psychological, other) and seriousness (serious vs non-serious).

The conditions of patients experiencing adverse effects due to the study interventions will be evaluated and referred for additional treatment if necessary.

Adherence to the PYM intervention will be assessed by recording the number of supervised PYM sessions attended and by using participant exercise diaries for HE, in which twice a week, duration and components of the prescribed exercises are recorded.

### Outcomes

As a primary outcome, the Fibromyalgia Impact Questionnaire (FIQ) will be used to assess participants’ functional disability before and after the intervention.^46^ The FIQ is a valid and reliable questionnaire with a total score ranging between 0–100 and measuring overall impact, including symptom severity, functionality, work difficulties, morning fatigue, depression, anxiety and general well-being rated by individuals with fibromyalgia. Higher scores indicate a greater impact of symptoms.^47^

Secondary outcomes will be measured using the following tools, with the following parameters being collected: Central Sensitisation Inventory (CSI),^48^ 20-item Multidimensional Fatigue Index (MFI-20),^49 50^ Pittsburgh Sleep Quality Index (PSQI),^51^ Perceived Stress Scale (PSS)^52^ and Visual Analogue Scale (VAS) for current, average and worst pain intensity of the participants over the last week.^53^

As a secondary outcome, changes in the autonomic nervous system will also be objectively recorded by measuring HRV. It will be measured and recorded with a Polar H10 (Polar Electro UK, Warwick, UK) chest strap using the validated Elite HRV mobile application, which has ease of use, reliability, data recording, storage, analysis and export features.^54^,^56^ The Polar H10 chest strap is CE certified and meets the EU requirements for non-invasive physiological data measurements. Participants will be requested to sit in silence for a period of 5 min, after which a 5-minute recording will be made for the purpose of analysis. During the HRV recording, participants will remain seated and quiet, with no physical activity. Breathing rate and depth will not be controlled. With the Polar H10 chest strap device, minimum-maximum-average Heart Rate (HR), HRV parameters: RMSSD (Square Root of the Mean of the Sum of the Squares of the Differences between RR Intervals), SDNN (Mean and SD of RR Intervals), total power, mean R-R interval, HF (High Frequency), LF (Low Frequency), LF/HF, (with their log-transformed indices lnRMSSD, lnHF, lnLF) will be collected and recorded in the Elite HRV application and manually entered into REDCap system.

Further assessments, as part of the initial measurement and questionnaire administration, demographic data of the participants will be collected. These data include age, sex, marital status, educational status, working status, duration of fibromyalgia and any relevant health conditions (other than exclusion criteria).

### Participants timeline

**Figure 1:**
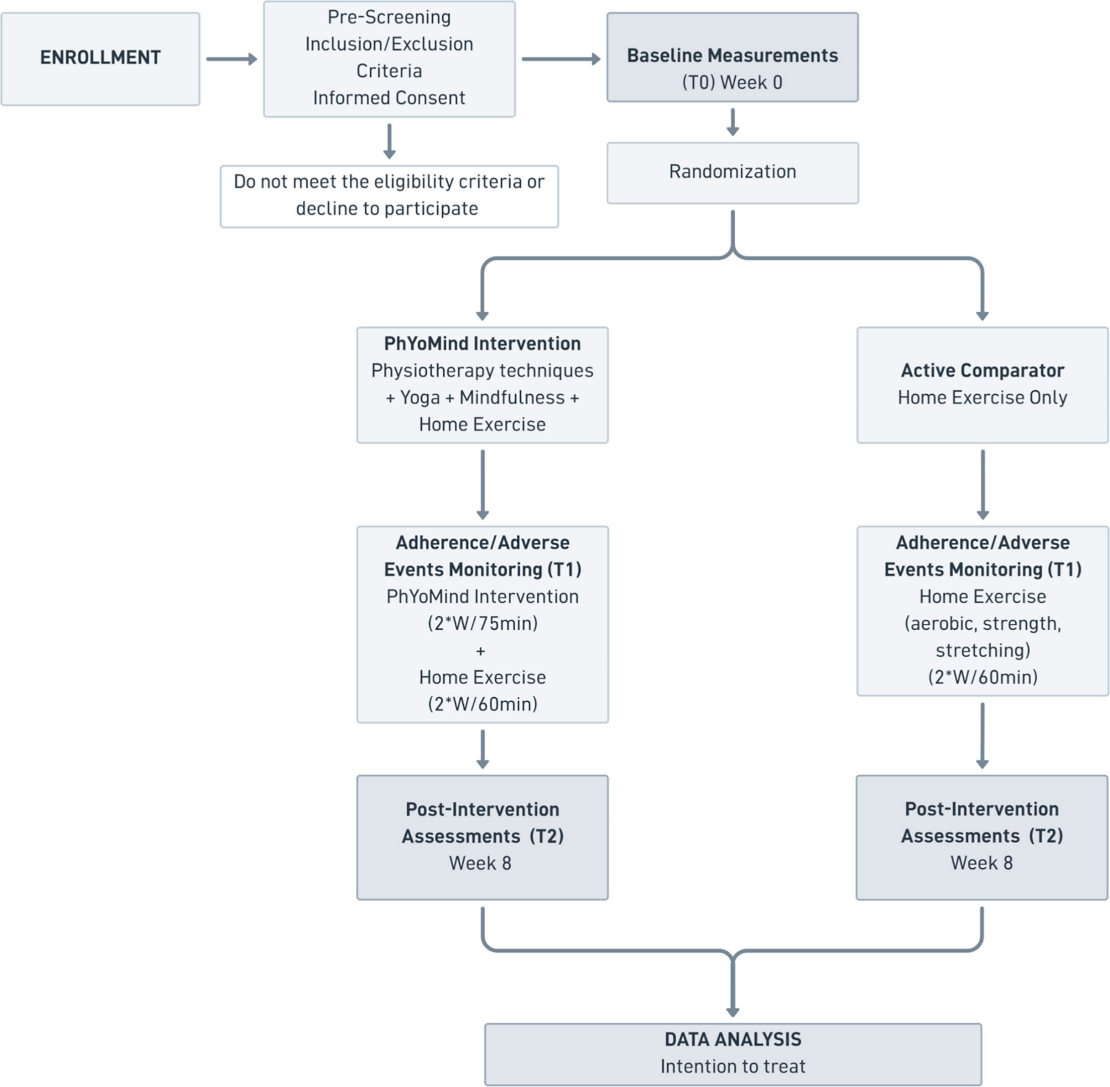
Participant flow diagram.

The study duration for each participant is approximately 10 weeks, and due to the two-cohort design, there is a possibility of additional waiting time. Outcome assessments will be conducted at two main time points: at baseline (T0), before randomisation and prior to the first intervention session, and at post-intervention (T2), in the week following completion of the 8-week programme. In both groups, adherence to the intervention and adverse events will be monitored from week 0 (T1) onwards and evaluated every 2 weeks during the 8-week period at weeks 2, 4, 6 and 8 as shown in figure 1. Participant flow diagram is detailed in table 2.

**Table 2:** Participant timeline: Schedule of enrolment, assessments and interventions

	Trial period		
	Enrolment/ baseline measurement	Initiation of the interventions Week 0	Post-intervention measurement Week 8
Timepoint	T_0_	T_1_	T_2_*
Enrolment:			
Eligibility criteria Phone-screening	X†		
Informed consent	X†		
Sociodemographic and clinical history	X†		
Invitation to the study centre	X†		
Inclusion/exclusion criteria	X ‡		
Diagnostic confirmation	X ‡		
Official inclusion of participants	X ‡		
Randomisation	X ‡		
Assessments:			
Fibromyalgia Impact Questionnaire (FIQ)	X‡		X
Central Sensitisation Inventory (CSI)	X‡		X
Heart Rate Variability (HRV)	X‡		X
Visual Analogue Scale (VAS)	X‡		X
Multidimensional Fatigue Index (MFI-20)	X‡		X
Pittsburgh Sleep Quality Index (PSQI)	X‡		X
Perceived Stress Scale (PSS)	X‡		X
Adverse events		X§ 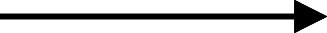	X§
Adherence		X§ 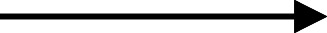	X§
Intervention/comparator:			
Intervention¶		X 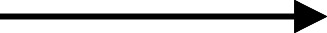	X
Comparator**		X 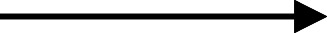	X

* Second measurement appointment at study centre.

† Before the first appointment.

‡ First appointment at study centre.

§ Adverse events and adherence (continuous monitoring; exercise diary review at weeks 2/4/6/8 covering the preceding interval).

¶ PhYoMind intervention (physiotherapy techniques+yoga+mindfulness) and home exercise programme.

** Only home exercise programme.

### Sample size estimation

Sample size was estimated using G*Power (V.3.1.9.7; Franz Foul, University of Kiel, Germany). The sample size was calculated based on a Repeated Measures Analysis of Variance (ANOVA), according to effect estimates from previous studies using our primary outcome, the Revised Fibromyalgia Impact Questionnaire.^57^ A minimum total number of participants of 32 is required to achieve 95% power with a type I error of α=0.05, for an expected effect size f=0.675 (d=1.35). Taking into account an expected dropout rate of 20%, the required sample size for our study is 40 participants, with 20 individuals per group.

### Randomisation and blinding

The block randomisation method with randomly varying block sizes between 2 and 8^58^ will be used to ensure an unbiased and random allocation of participants into the two treatment groups: Intervention group and control group (1:1). A study team member (AAl) who is not involved in participant recruitment, outcome assessment or intervention delivery generated the random allocation sequence using the R statistical software (V.4.5.0, R Core Team 2025) and the package *blockrand*, V.1.5.^59^ The full randomisation list is stored in a secure file inaccessible to study team members responsible for enrolment, assessment or intervention delivery. Immediately after baseline measurement, randomisation will be carried out using the REDCap system^60 61^ and participants will then be informed of their group allocation prior to the first intervention appointment.

The outcome assessor as well as the statistician performing the data analysis will be blinded to group allocation. Due to the nature of the interventions, participants and the study physician delivering the PYM sessions cannot be blinded.

To maintain blinding, group labels will be coded (eg, Group A/Group B) in the dataset that will be provided to the statistician, and outcome assessments will be scheduled and conducted separately from the intervention sessions. Participants will be requested not to disclose details of their assigned intervention during assessments, and outcome assessors will be instructed not to ask about group allocation. Unblinding of the outcome assessor or statistician will occur only in exceptional circumstances when knowledge of treatment allocation is essential for participant safety. The principal investigator will request unblinding from the data manager (who holds the allocation file), if it’s necessary and the reason and timing will be documented.

### Data collection and management

Outcome measurements will be carried out before and after the 8-week programme; adverse effects and adherence will be monitored every 2 weeks. Data collection and all validated questionnaires will be administered by the outcome assessors: Baseline eligibility and ACR-criteria confirmation will be performed by ANVK, a study physician; and (HSK), a physiotherapist and doctoral researcher who will conduct the primary and secondary outcome assessments at both baseline and post-intervention. Assessments will take place at baseline and at week 8 of the study which includes the confirmation of eligibility/visit procedures, HRV measurement and completion of questionnaires. To ensure consistency across participants and visits, a structured assessment procedure will be employed, encompassing both written instructions and a checklist. For example, instructions regarding points to keep in mind regarding the HRV prior to the measurement appointment will be provided to participants via email and telephone (eg, avoiding alcohol/coffee, smoking, intense exercise and heavy meals before HRV assessment). A checklist will be prepared containing instructions for the outcome assessor to follow, written in the same order and using the same wording during the measurements. Participants will primarily complete questionnaires electronically on tablet via REDCap; paper forms will be available if required and entered into REDCap by the outcome assessors. HRV measurements will be recorded under standardised conditions (quiet room, seated position, after a brief rest) and stored in REDCap. Should any artefacts requiring more than 5% correction be detected, whether due to unintended movement or speech during HRV measurement, the procedure will be repeated. To promote data quality and ensure higher adherence, appointments will be scheduled flexibly, reminder calls or e-mails will be used when necessary, and a contact person will be available during the study. For participants who discontinue or deviate from the intervention protocols, all feasible outcome data at week 8 will be collected, provided that informed consent is maintained.

Only anonymised results will be reported, and study data will be retained for up to 10 years in line with institutional and legal requirements before being deleted or irreversibly anonymised. Participants may withdraw their consent at any time without disadvantage. No further data will then be collected, but any pseudonymised data already collected may continue to be used for analysis, unless patients explicitly request their deletion.

The study team will monitor the conduct of the study, participant recruitment, informed consent procedures, protocol compliance, adverse events and data integrity through regular internal reviews. Monitoring will be carried out continuously throughout the study, and a formal review will take place at regular study team meetings. As this is a monocentric, low-risk, non-pharmacological study, no separate external monitoring body has been established.

### Statistical analysis

The data obtained during the study will be analysed using the statistical software R, V.R-4.3.3 for Windows. Descriptive statistics including mean, SD, median, etc, will be calculated for demographic and clinical characteristics in both groups. Repeated Measures ANOVA (rmANOVA) will be used to analyse the effect of time, intervention group and their interaction on primary and secondary outcomes. Time will be considered as the within-subject factor and group will be considered as the between-subject factor. Effect sizes and group differences will be reported subsequently. The significance level will be p<0.05.

To ensure the robustness and validity of the statistical analysis, missing data will be addressed using multiple imputation. All randomised participants will be included in the main analyses according to the intention-to-treat principle and will be analysed in the groups to which they were originally allocated. Serious and non-serious adverse events and drop-outs during the intervention period will be summarised and compared between groups using absolute and relative frequencies. Given the relatively limited sample size, no formal mediation analysis is planned as part of the confirmatory or secondary statistical analyses. Associations between fibromyalgia severity, perceived stress and clinical outcomes may be explored descriptively or in exploratory regression models, but these analyses will be considered hypothesis-generating and interpreted with caution. No formal interim analyses are planned, and no predefined stopping rules for efficacy or futility have been established.

### Patient and public involvement statement

No patients or members of the public are involved in the design, conduct, reporting of this study. Published results will be shared with interested participants.

## Discussion

The aim of this study is to investigate whether the PYM intervention, that integrates various techniques used in physical therapy with yoga and mindfulness, has an additional or synergistic effect when added to a home-exercise programme in individuals with fibromyalgia. Accordingly, effects will be compared between PYM plus HE and HE alone.

This study is addressing a significant evidence gap in this area. Investigating the effectiveness of a comprehensive, multimodal, integrated fibromyalgia intervention that may provide additional and potentially synergistic benefits beyond those achieved with HE alone could offer a new perspective to the existing literature.

This study is limited by a short-term follow-up (8 weeks). Although outcome assessors and statisticians will be blinded, blinding participants is not possible due to the nature of the intervention. In light of this research, future multi-arm trials could include additional comparator groups to isolate the contribution of individual PYM components. Nonetheless, our study may serve as a starting point for future trials evaluating long-term outcomes of the proposed PYM intervention and it has the potential to generate increased interest in the development of integrative and complementary, non-pharmacological, sustainable and cost-effective interventions.

### Ethics approval

Ethics approval was granted from the Ethics Committee at the Medical Faculty of Eberhard Karls Tuebingen University and at the University Hospital of Tuebingen (260/2025BO2) and the study was registered at ClinicalTrials.gov (NCT07145788). Written informed consent will be obtained from all participants by the study physician (ANVK) who will provide oral and written information about the study and answer any participant questions prior to consent. The ethics committee will be informed of any deviations from the protocol, serious or unknown adverse events, and new findings that indicate a risk to the safety of the participants. The trial sponsor and funders had no role in study design, study conduct, data analysis, interpretation of findings, or the decision to publish.

## Supplementary material

10.1136/bmjopen-2026-120248 online supplemental file 1

10.1136/bmjopen-2026-120248 online supplemental file 2

10.1136/bmjopen-2026-120248 online supplemental file 3

10.1136/bmjopen-2026-120248 online supplemental file 4

10.1136/bmjopen-2026-120248 online supplemental file 5

10.1136/bmjopen-2026-120248 online supplemental file 6

## Data Availability

Data are available upon reasonable request.
