# Supplementary material for: Physiotherapy-integrated yoga and mindfulness plus home exercise versus home exercise alone for individuals with fibromyalgia syndrome (PhYoMind): study protocol of a randomised controlled clinical trial
Source: BMJ Open. 2026 Jul 6;16(7):e120248. doi: 10.1136/bmjopen-2026-120248 (PMC13343093; doi:10.1136/bmjopen-2026-120248)
Supplement: online supplemental file 1 [file bmjopen-16-7-s001.pdf]

Universitätsklinikum Tübingen

Institute for General Medicine and Interprofessional Care

Chair for Research on Complementary Medicine

5 Osianderstr.

72076 Tübingen

Prof. Holger Cramer, M.D.

holger.cramer@med.uni-tuebingen.de

Phone: 0711/8101-2831

Patient Consent Form

Physiotherapy-Integrated Yoga and Mindfulness plus Home Exercise versus Home Exercise alone for Individuals with Fibromyalgia Syndrome (PhYoMind): A Randomized Controlled Clinical Trial

Patient :____

(Last Name, First Name)

Date of Birth: __ __ . __ __ . __ __ __ __

The participant was informed about the clinical study by

____

(Investigating Physician)

and covered the following points:

- Nature and objectives of the clinical trial
- Nature and implementation of the yoga treatment and home exercises, including potential effects and side effects
- Nature and implementation of the examination methods (questionnaire, heart rate variability), including benefits and risks
- Information regarding the **lack of** accident insurance
- Information regarding the **lack of** participant insurance
- Right to withdraw from the clinical trial
- Information on data protection: Documentation, sharing, and publication of participant data will be conducted in pseudonymized form
- We were provided with a copy of the written subject information sheet and the signed informed consent form

I agree to participate in the PhYoMind study and confirm that the informational discussion covered the points listed above. I am aware that I may withdraw my consent to participate in this clinical trial at any time without providing a reason and that this will have no adverse effects on my further treatment. All my questions regarding the study have been answered. **The withdrawal must be addressed to the principal investigator, Prof. Dr. Holger Cramer** (Osianderstr. 5, 72076 Tübingen, Tel.: 0711/8101-2831, Email: holger.cramer@med.uni-tuebingen.de).

______________________________ ______________________________

Place, Date Patient's signature

______________________________ ______________________________

Place, Date Investigator's signature
