# Supplementary material for: Physiotherapy-integrated yoga and mindfulness plus home exercise versus home exercise alone for individuals with fibromyalgia syndrome (PhYoMind): study protocol of a randomised controlled clinical trial
Source: BMJ Open. 2026 Jul 6;16(7):e120248. doi: 10.1136/bmjopen-2026-120248 (PMC13343093; doi:10.1136/bmjopen-2026-120248)
Supplement: online supplemental file 2 [file bmjopen-16-7-s002.pdf]

Universitätsklinikum Tübingen

Institut für Allgemeinmedizin und interprofessionelle Versorgung

Professur zur Erforschung komplementärmedizinischer Verfahren

Osianderstr. 5

72076 Tübingen

Univ.-Prof. Dr. Holger Cramer

holger.cramer@med.uni-tuebingen.de

Tel.: 0711/8101-2831

Einwilligungserklärung zur Teilnahme in der Studie:

Physiotherapie-integriertes Yoga und Achtsamkeit plus Heimübungen im Vergleich zu reinen Heimübungen bei Personen mit Fibromyalgie-Syndrom (PhYoMind): Eine randomisierte kontrollierte klinische Studie

PatientIn:____

(Name, Vorname)

Geb.-Datum: __ __ . __ __ . __ __ __ __

Die Aufklärung des Probanden über die klinische Studie erfolgte durch

____

(Prüfarzt)

und erstreckte sich auf folgende Punkte:

- Art und Zielsetzung der klinischen Studie
- Art und Durchführung der Yogabehandlung und der Heimubungen einschließlich der möglichen Wirkungen und Nebenwirkungen
- Art und Durchführung der Untersuchungsmethoden (Fragebogen, Herzfrequenz-Variabilität) einschließlich Nutzen und Risiken
- Information über die **nicht bestehende** Wegeunfallversicherung
- Information über die **nicht bestehende** Probandenversicherung
- Recht auf Rücktritt von der klinischen Studie
- Informationen zum Datenschutz: Dokumentation, Weitergabe und Veröffentlichung der Probandendaten erfolgen in pseudonymisierter Form
- Eine Kopie der schriftlichen Probandeninformation und der unterschriebenen Einverständniserklärung wurden uns ausgehändigt

Ich bin damit einverstanden, an der PhYoMind-Studie teilzunehmen und bestätige, dass sich das Aufklärungsgespräch auf die oben angeführten Punkte erstreckte. Mir ist bekannt, dass ich jederzeit ohne Angabe von Gründen die Einwilligung zur Teilnahme an dieser klinischen Prüfung widerrufen kann und dass dies keine nachteiligen Auswirkungen auf meine weitere Behandlung hat. Alle meine Fragen zur Studie wurden beantwortet. **Der Widerruf ist zu richten an den Studienleiter Prof. Dr. Holger Cramer** (Osianderstr. 5, 72076 Tübingen, Tel.: 0711/8101-2831, Mail: holger.cramer@med.uni-tuebingen.de).

______________________________ ______________________________

Ort, Datum Unterschrift des Patienten

______________________________ ______________________________

Ort, Datum Unterschrift des Prüfarztes
