# Supplementary material for: Physiotherapy-integrated yoga and mindfulness plus home exercise versus home exercise alone for individuals with fibromyalgia syndrome (PhYoMind): study protocol of a randomised controlled clinical trial
Source: BMJ Open. 2026 Jul 6;16(7):e120248. doi: 10.1136/bmjopen-2026-120248 (PMC13343093; doi:10.1136/bmjopen-2026-120248)
Supplement: online supplemental file 3 [file bmjopen-16-7-s003.pdf]

Set Intention

1. Set Intention Section

Intention: “I move my hips with curiosity and kindness, building strength and mobility in a way my nervous system can trust”
Today we’ll practice Ahimsa, a yoga principle meaning non-violence, treating yourself with care rather than force. With fibromyalgia, the nervous system may become protective and encourage guarding. Our goal isn’t to “open” the hips by pushing; it’s to support a sense of safety in movement. We’ll use slow, graded motions and steady breath to explore range without provoking alarm. As we practice, check three signals: breath (is it flowing?), face/jaw (are they tightening?), and hip/belly tone (is there bracing?). If you notice breath-holding, clenching, or pain that feels sharp or is building, that’s not failure, it’s information. Soften, choose a simpler option, and stay within a workable range.

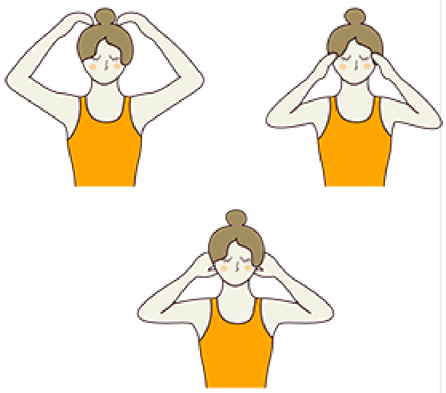

2. Head Tapping Exercise Close Up

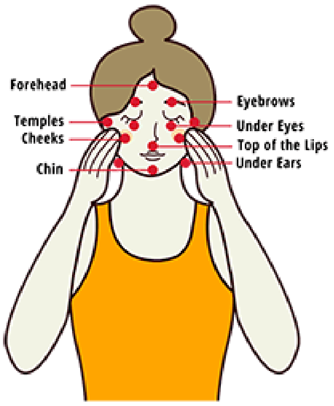

3. Face Tapping Exercise Close Up

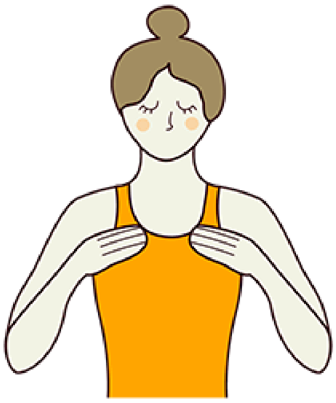

4. Chest Tapping Exercise Close Up

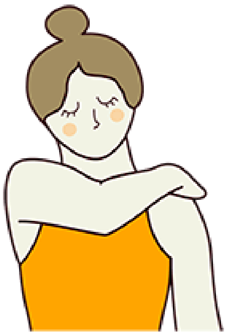

5. Shoulder Tapping Exercise Close Up

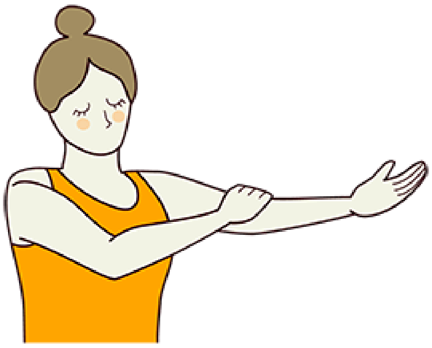

6. Arm Tapping Exercise Close Up

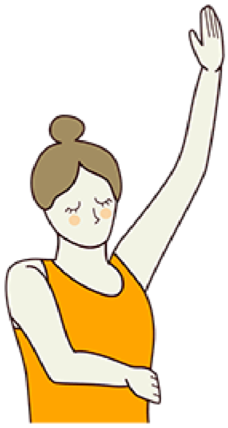

7. Side Of Body Tapping Exercise Close Up

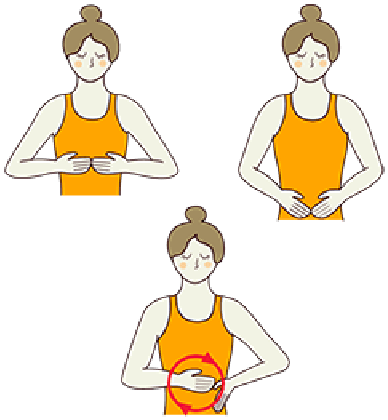

8. Belly Tapping Exercise Close Up

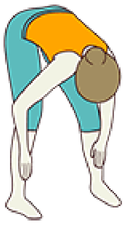

9. Legs Tapping Exercise

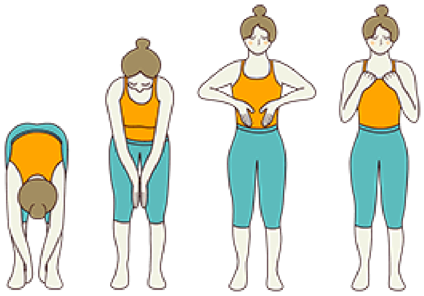

10. Tracing Kidney Meridian Movement

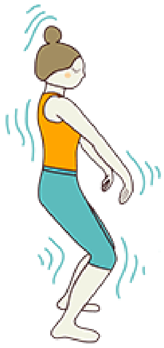

11. Shaking Therapy Pose

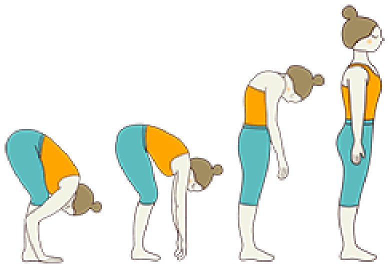

12. Standing Spinal Roll Up Pose Flow

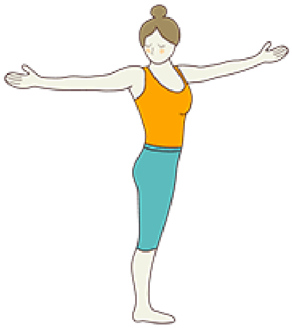

13. Mountain Pose Twist Arms Shoulder Level Spread Out • Tadasana Twist Arms Shoulder Level Spread Out

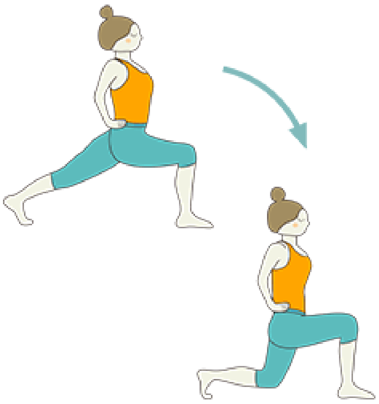

14. Crescent High Lunge Pose Arms On Hips Flow • Ashta Chandrasana Arms On Hips Vinyasa

"**Sciatic Nerve Gliding**" has been integrated. Chair can be used as a support.

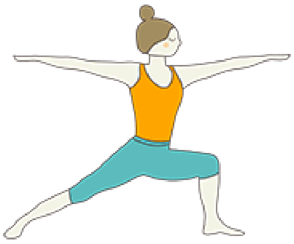

15. Warrior Pose Ii • Virabhadrasana Ii

"**Median Nerve Gliding**" has been integrated. Chair can be used as a support.

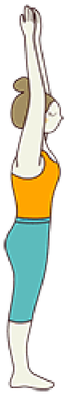

16. **Mountain Pose Raised Hands Head Straight** • Tadasana Urdhva Hastasana Head Straight

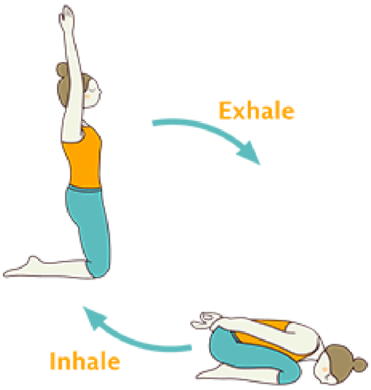

17. **Kneeling Pose Child Pose Flow** • Utthita Vajrasana Balasana Vinyasa

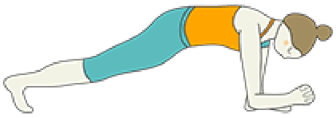

18. **Lizard Pose** • Utthan Pristhasana

Simple variations (e.g., knees on the floor) can be implemented.

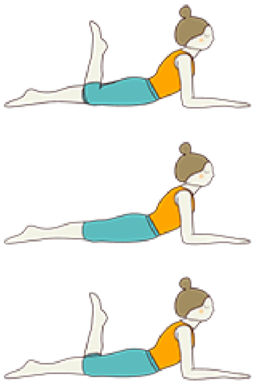

19. **Sphinx Pose Variation Leg Movement** • Salamba Bhujangasana Pada Movement

"**Femoral Nerve Gliding**" has been integrated.

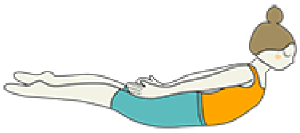

20. **Locust Pose** • Salabhasana

Simple variations (e.g., head/legs on the floor) can be implemented.

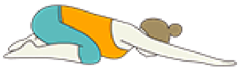

21. **Wide Child Pose** • Prasarita Balasana

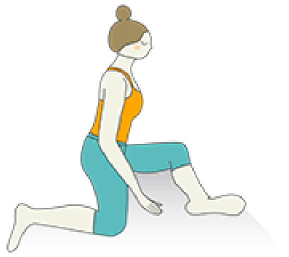

22. **Half Pigeon Pose Variation** • Ardha Kapotasana Variation

"Hip Internal/External Rotation Mobilization + PNF Contract-Relax/Antagonist Contract Techniques" have been integrated

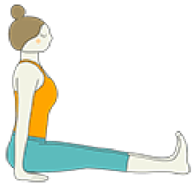

23. **Staff Pose** • Dandasana

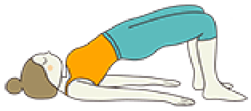

24. **Bridge Pose** • Setubandha Sarvangasana

"PNF Contract-Relax Antagonis Contract technique" has been integrated (Bolster/soft pillow under the sacrum)

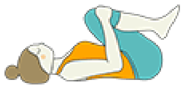

25. **Wind Release Pose** • Pawanmuktasana

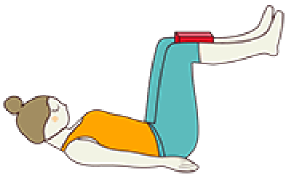

26. **Half Boat Pose Block Between Bent Knees** • Ardha Navasana Block Between Bent Knees

"PNF Contract-Relax/Antagonist Contract Techniques" have been integrated

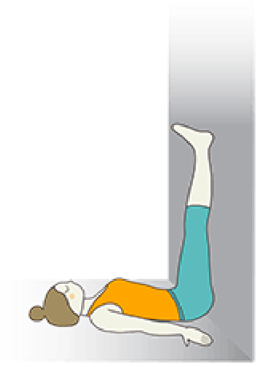

27. **Legs Up The Wall Pose** • Viparita Karani Wall

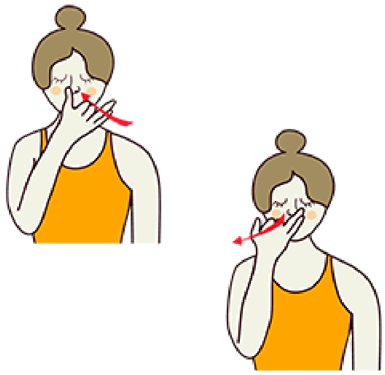

28. **Alternate Nostril Breathing Close Up** • Nadi Shodhana Pranayama

"Left Nostril Breathing" is implemented if it's possible.

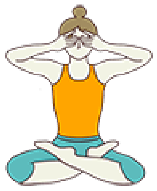

29. **Bumble Bee Breath** • Bhramari Pranayama

Easy sitting pose, maybe with bolster, or lying down, eyes can be opened/closed. Ears can be closed gently with thumbs (optional)

Breath Awareness

30. **Breath Awareness Section**

Body Observation

31. **Body Observation Section**

Observation of Thoughts

32. **Observation Of Thoughts Section**

Silence

33. **Silence Section** • Maun Section

Mindfulness Practice

34. Mindfulness Practice Section

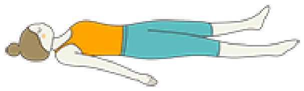

35. Corpse Pose • Shavasana

Isometrically Squeeze

36. Isometrically Squeeze Section

Post-Isometric Relaxation on Shavasana
Feet/soles → calves → thighs → glutes → belly → chest/back → shoulders → arms/hands → neck → jaw/tongue/face → scalp.

Body Scan

37. Body Scan Section

Deep Rest

38. Deep Rest

Finishing Sequence

39. Finishing Sequence Section
