# Supplementary material for: Physiotherapy-integrated yoga and mindfulness plus home exercise versus home exercise alone for individuals with fibromyalgia syndrome (PhYoMind): study protocol of a randomised controlled clinical trial
Source: BMJ Open. 2026 Jul 6;16(7):e120248. doi: 10.1136/bmjopen-2026-120248 (PMC13343093; doi:10.1136/bmjopen-2026-120248)
Supplement: online supplemental file 4 [file bmjopen-16-7-s004.pdf]

RBIM

Robert Bosch Centrum für Integrative Medizin und Gesundheit

Home Exercises

Universitätsklinikum Tübingen

These exercises should be performed for a total of **8 weeks on 2 days per week**.

The exercises should not be performed on consecutive days, but with a **one-day break** in between.

During training, you should monitor your breathing rate and your body's general reactions. Make sure you feel comfortable during training and remember to rest when necessary.

Listen to your body and take it step by step. If you have any problems, please contact the study team.The training should last an average of **60 minutes.**

1 Walking 30 min

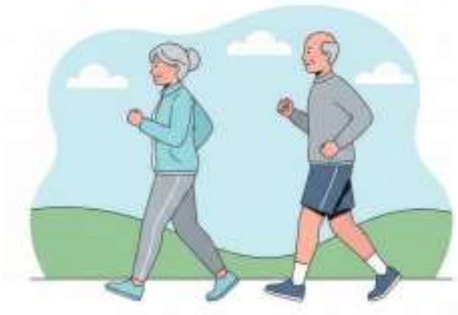

2 Plank-Exercise

Position 1

Position 2

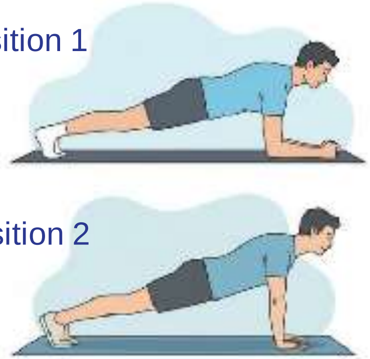

2 Plank-Exercise

Easy version

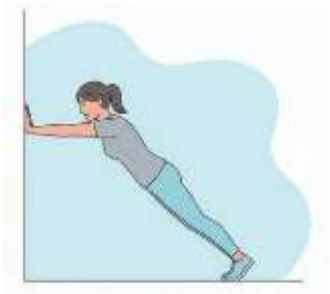

1 Start at a slow pace for a few minutes. Then continue at a pace where you are slightly out of breath when talking, but can still hold a conversation easily. In the last few minutes, return to a slow pace and finish your walk.

2 For the plank, support yourself on your hands and toes, as shown. Your body should form a straight line. Your hands should be under your shoulders, and your stomach and buttocks should be firm. If you experience discomfort in your hands, move into the forearm support position by placing your elbows under your shoulders. Breathe in and out calmly and hold for about 20–30 seconds or as long as you can. Repeat this 3 times and increase the duration of the exercise. The easier version (against the wall): Place your hands shoulder-width apart on the wall (at shoulder height). Walk your feet back until your body forms a straight line. Tighten your stomach, slightly tense your buttocks, and keep your head in line with your spine. Hold for 10–30 seconds, 3 times.

KOM | 02.2026

Bosch Health Campus

der Robert Bosch Stiftung

3 Exercise to strengthen the back

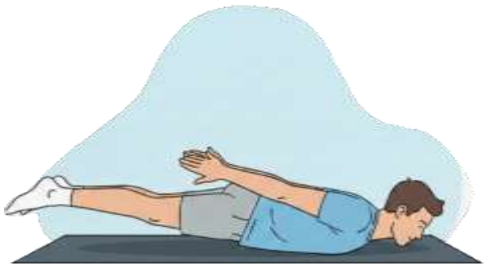

3 Exercise to strengthen the back

Easy version

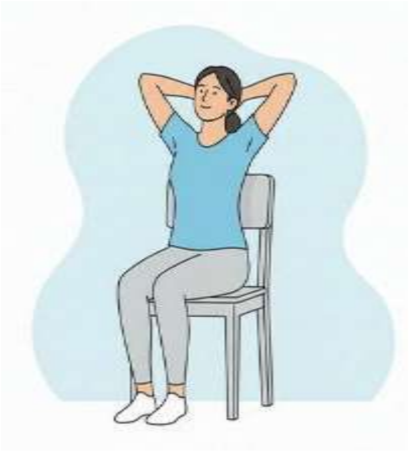

4 Exercise to strengthen the arms

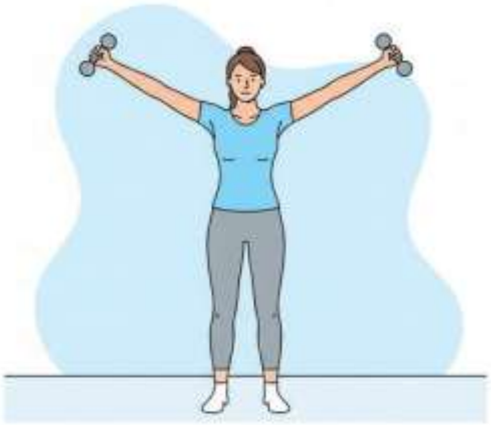

3 Extend your arms out to the sides of your body, as shown in the picture. As you inhale, lift your upper body, arms, and legs off the floor. Hold for 2–3 seconds, then slowly lower your upper body, arms, and legs back to the floor as you exhale. Perform 3 sets of 8-12 repetitions. The easier version (sitting): Sit upright on a chair. Place your hands behind your head with your elbows wide. Lift your sternum and bring your shoulder blades together at the back, then slowly return to the neutral sitting position. Perform, 2-3 sets of 8-12 repetitions.

4 Hold a tolerable weight in each hand and start with your arms at your sides. Raise your straight arms to a 45° angle until they are horizontal, as shown in the picture. Make sure your thumbs are pointing upwards. Perform 3 sets of 8-12 repetitions. This exercise is ideal for strengthening the muscles in your arms and shoulders.

KOM | 02.2026

5 Stretching the back of the legs and the spine

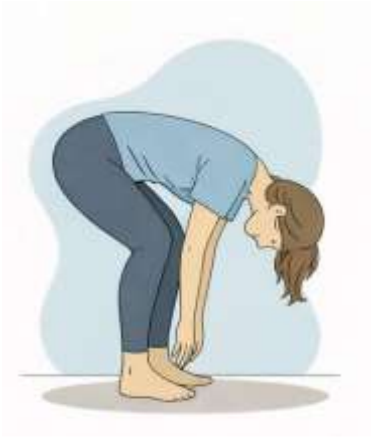

5 Stretching the back of the legs and the spine

Easy version

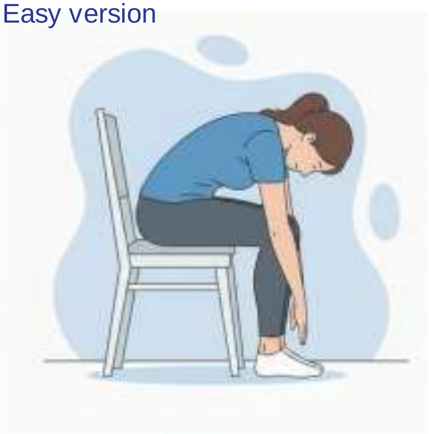

6 Squat Exercise

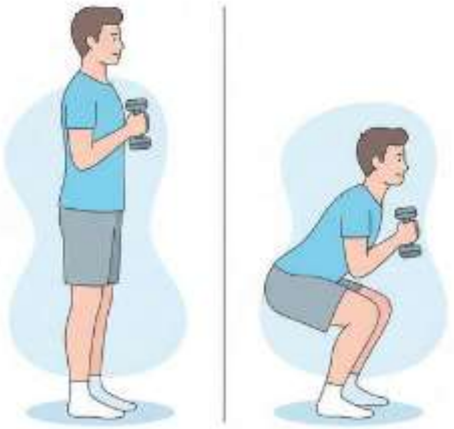

5 Place your feet hip-width apart. Lower your upper body forward with your knees slightly bent and let your hands slide toward the floor. Let gravity pull you down slowly. Hold for 30 seconds and then slowly straighten up again, vertebra by vertebra. Repeat 1–2 times. The easier version (on a chair): Place your feet hip-width apart, knees and hips at a 90° angle. Slowly lower your upper body forward and let your hands slide toward the floor. Hold for 30 seconds, then slowly straighten up, vertebra by vertebra. Repeat 1–2 times.

6 Place your feet hip-width apart and perform a squat with your back straight. Move backwards as if there were a chair behind you. Once you get the hang of it, you can hold weights in your hands to intensify the exercise. Perform this exercise 3 sets of 8-12 repetitions.

KOM | 02.2026

7 Stretching the front of the thighs

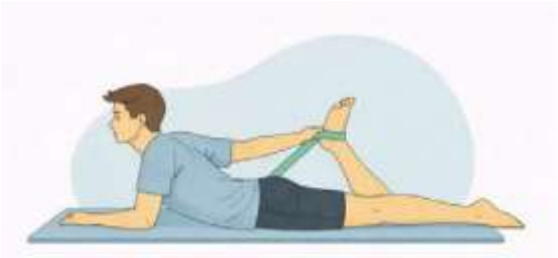

7 Stretching the front of the thighs

Easy version

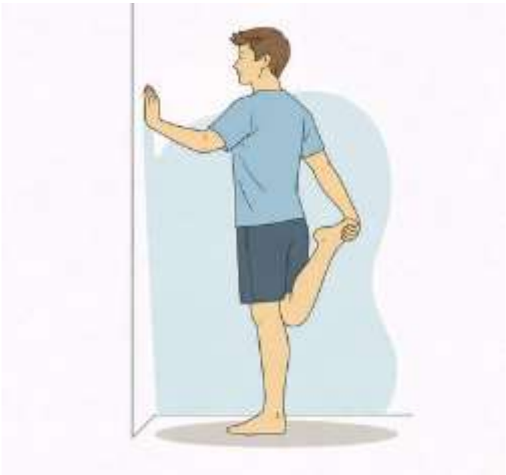

8 Stretching the chest muscles

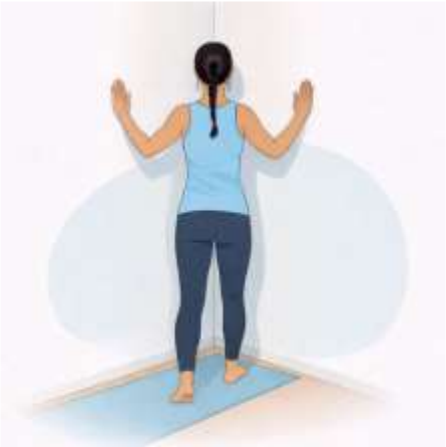

7 As shown in the first picture, get into a prone position. Bend one of your leg at the knee joint with the help of a towel, sheet or belt. Bring your heel closer to your buttocks and stretch the front of your thigh for 30 seconds. Perform the exercises 1-2 times on each side. The easier version of the exercise: Support yourself with one hand on the wall. Stand on one leg. Bend the other knee, grasp the foot/ankle on the same side and pull the heel towards your buttocks. Knees together, back neutral. Hold each side 1-2 times for 30 seconds.

8 Place your hands on the corner of the room and bend your elbows as shown in the picture. Place your feet a large step away from the wall. Then bend your chest towards the corner. Hold for 30 seconds gently and then relax. Repeat 1-2 times.

KOM | 02.2026

9 Neck Streching

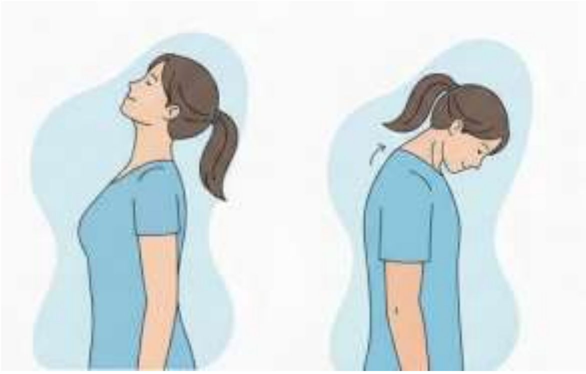

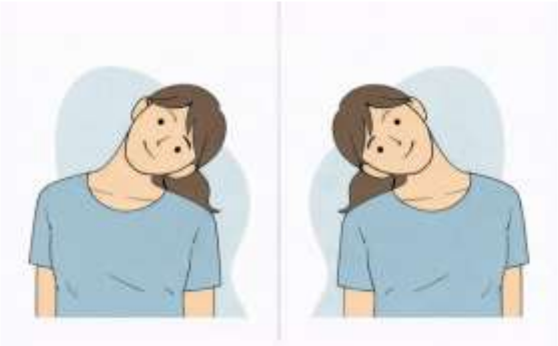

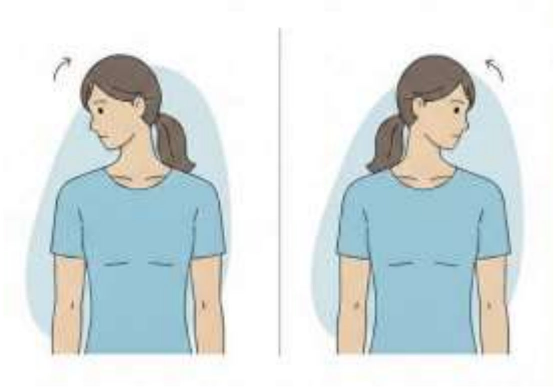

9 Perform the neck movements in sequence as shown in the illustrations. Hold each position for 30 seconds. If necessary, gently support the stretch with your hand. Breathe slowly and deeply and relax while performing these movements. Repeat the sequence 3 times.

KOM | 02.2026

Safety when performing the exercises

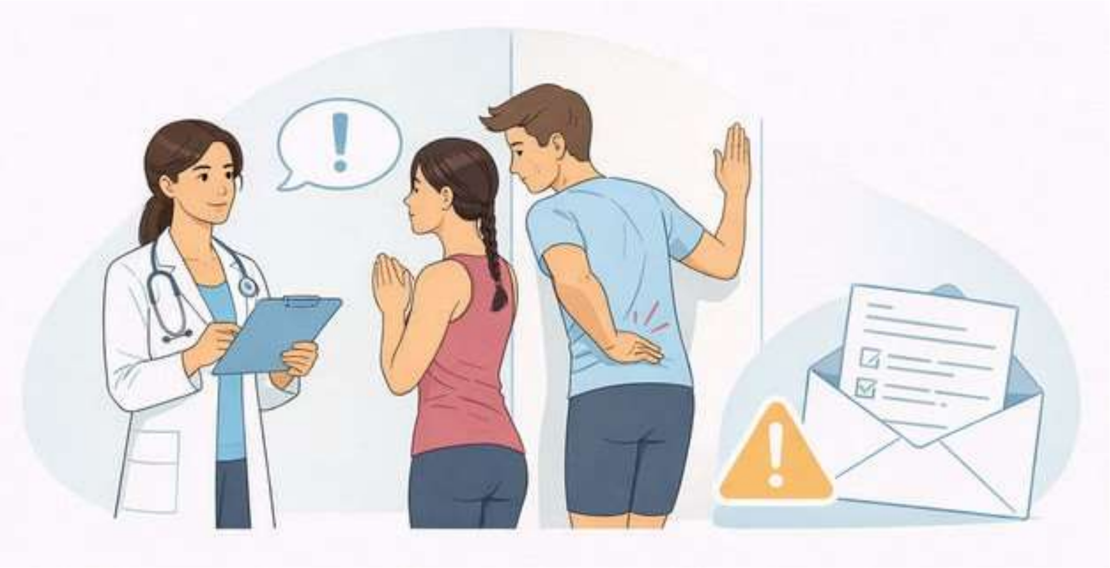

If you encounter any problems while performing the exercises, please inform the research team.

Every two weeks, you will receive a questionnaire by email from the research team asking about your participation in the exercises and any problems you may have encountered.

Bosch Health Campus GmbH
Robert Bosch Centrum für Integrative Medizin und Gesundheit

Auerbachstraße 112 | 70376 Stuttgart
Telefon +49 711 8101-7858
rbim.studien@bosch-health-campus.com
www.bosch-health-campus.com

The illustrations for the exercises were created with AI support (ChatGPT and Google Gemini). The study team reviewed the final materials for comprehensibility and safety.

KOM | 02.2026
