# Supplementary material for: Physiotherapy-integrated yoga and mindfulness plus home exercise versus home exercise alone for individuals with fibromyalgia syndrome (PhYoMind): study protocol of a randomised controlled clinical trial
Source: BMJ Open. 2026 Jul 6;16(7):e120248. doi: 10.1136/bmjopen-2026-120248 (PMC13343093; doi:10.1136/bmjopen-2026-120248)
Supplement: online supplemental file 6 [file bmjopen-16-7-s006.pdf]

PhYoMind Study – Control Group 2-Week Exercise Diary Questionnaire

Weeks: ……–…… Participant ID: ……

Instructions:

A total of 4 home exercise sessions were planned for the past 2 weeks (2 sessions per week, each lasting 60 minutes). Please answer the following questions about your home exercises during this period. Tick the answer that best describes your experience.

A. Participation

1. Over the past two weeks, 4 home exercise sessions were planned. How many of these sessions did you complete?

- ○ *0 out of 4*
- ○ *1 out of 4*
- ○ *2 out of 4*
- ○ *3 out of 4*
- ○ *4 out of 4*

B. Dose (duration of the session)

2. A total of 240 minutes of home exercises were planned for the past 2 weeks (e.g., 4 × 60 minutes).

How many minutes did you actually spend on home exercises in the past 2 weeks?

Answer: _________ minutes (0–240)

C. Overall adherence/difficulties

3. Did you have significant difficulties participating in any part of the home exercises over the last 2 weeks?

- ○ No
- ○ Yes

4. If "Yes": Which exercises did you often have difficulty doing?

(Please check all that apply.)

- ○ Walking
- ○ Plank exercise
- ○ Back strengthening exercise
- ○ Arm strengthening exercise
- ○ Squat exercise
- ○ Chest muscle stretch
- ○ Neck stretch
- ○ Back of the leg and spine stretch
- ○ Front of the thigh stretch

D. Adverse events and changes in symptoms

5. Have you had any new or significantly increased symptoms in the past 2 weeks that you think may be related to the home exercises?

- No
- Yes

6. If yes, or if you would like to provide us with more detailed information: Have you experienced any of the following physical symptoms during or immediately after your home exercises in the last 2 weeks?

(Multiple answers possible)

- Increased muscle or joint pain
- Dizziness or feeling unsteady when standing/walking
- Heart palpitations or significantly accelerated pulse
- Shortness of breath or tightness in the chest
- Headaches
- Nausea or stomach problems
- Muscle cramps or pronounced muscle fatigue
- Other physical complaints: ___________________________
