# Supplementary material for: Physiotherapy-integrated yoga and mindfulness plus home exercise versus home exercise alone for individuals with fibromyalgia syndrome (PhYoMind): study protocol of a randomised controlled clinical trial
Source: BMJ Open. 2026 Jul 6;16(7):e120248. doi: 10.1136/bmjopen-2026-120248 (PMC13343093; doi:10.1136/bmjopen-2026-120248)
Supplement: Supplementary file 7 [file bmjopen-2026-120248.reviewer_comments.pdf]

PEER REVIEW HISTORY

BMJ Open publishes all reviews undertaken for accepted manuscripts. Reviewers are asked to complete a checklist review form and are provided with free text boxes to elaborate on their assessment. These free text comments are reproduced below.

ARTICLE DETAILS

Title (Provisional)

Physiotherapy-Integrated Yoga and Mindfulness plus Home Exercise versus Home Exercise alone for Individuals with Fibromyalgia Syndrome (PhYoMind): A Study Protocol of a Randomized Controlled Clinical Trial

Authors

Sarak Kucukosmanoglu, Hazal; Aytar, Aydan; Bilc, Mirela-Ioana; Anheyer , Dennis; N. V. Kieninger, Alexandra; Alaze, Anita; Cramer, Holger

---

VERSION 1 - REVIEW

**Reviewer**	**1**
**Name**	**Jiang, Liming**
**Affiliation**	**Shanghai University of Traditional Chinese Medicine, Shanghai Seventh People's Hospital**
**Date**	**23-Apr-2026**
**COI**	**None**

Overview

This protocol primarily observes the efficacy of a PhYoMind treatment plan for fibromyalgia. While it has some clinical applicability, it does not explain the rationale for combining these treatment methods and lacks novelty. See below for specific comments.

Major issue

1. Using home exercise alone as a control group seems unreasonable, as it is not a high-level evidence-based treatment recommended in fibromyalgia treatment guidelines.
2. This study was designed as a single-blind trial, yet it also mentions that both the outcome assessors and statisticians were blinded, which is contradictory. According to the latest RCT writing guidelines, the blinding method includes four groups of people. Please explain in detail the specific subjects and procedures of the blinding method.

3. Please add the assessor's qualifications and a detailed description of the assessment process.

4. This study used repeated measures ANOVA to estimate the sample size, but the effect size was calculated using "d" instead of "f" or "η^2". Please confirm this.

5. Please provide a detailed description of the innovativeness of this comprehensive therapy.

Minor issue

1. Please add a simple recruitment flowchart.
2. The inclusion criteria should include requirements for the duration of fibromyalgia and the initial pain level.
3. What is the basis for the treatment prescriptions set in the intervention group, and is there any literature or other evidence to support it?

VERSION 1 - AUTHOR RESPONSE

Reviewer: 1
Mr. Liming Jiang, Shanghai University of Traditional Chinese Medicine
Comments to the Author:
Overview
This protocol primarily observes the efficacy of a PhYoMind treatment plan for fibromyalgia. While it has some clinical applicability, it does not explain the rationale for combining these treatment methods and lacks novelty. See below for specific comments.
Major issue
1. Using home exercise alone as a control group seems unreasonable, as it is not a high-level evidence-based treatment recommended in fibromyalgia treatment guidelines.

Thank you for this comment. We agree that the comparator should reflect evidence-based care and that the choice of the comparator is critical. There are various guidelines applicable in the context of FMS in Germany. One of these is the EULAR (European Alliance of Associations for Rheumatology) guideline, which is applicable within the European context and recommends various non-pharmacological treatments for FMS, such as exercise, mind-body therapies, mindfulness, massage, biofeedback, etc. (https://pubmed.ncbi.nlm.nih.gov/27377815/). However, exercise is the only non-pharmacological treatment that is strongly recommended (strong for 100% agreement) based on high-quality evidence from reviews and meta-analyses. Specifically, aerobic and strength exercise are mentioned as type of exercises. In addition, one of the national guideline for FMS in Germany emphasizes the importance of aerobic, strength and flexibility training being an essential part for FMS treatment, and these are strongly recommended based on high-quality evidence (https://register.awmf.org/de/leitlinien/detail/145-004). Taking into account the ACSM's (American College of Sports Medicine) exercise prescription and adherence

recommendations for FMS, we considered each of these guidelines when developing the exercise prescriptions for the home exercise group (https://www.frontiersin.org/journals/physiology/articles/10.3389/fphys.2024.1413038/full). Therefore, the control group includes some of the strongest evidence that can be found across these guidelines. The combination of the different guidelines was used to combine all the specific aspects of exercise, such as duration, frequency, intensity, in the home exercise program. (Because not all guidelines mention all these aspects. In order to avoid overloading FMS patients, a simple, guideline-based exercise program has been designed to take all these factors into account.

In conclusion, the home exercise program comprises aerobic exercise (twice a week, 60 minutes), strength training (twice a week, using weights or body weight, 3 sets of 8–12 repetitions) and flexibility exercises (twice a week, 30 seconds, 1–2 repetitions). To better address your concerns, we have expanded on the introduction section in greater detail to clarify. The changes are visible in the revised manuscript with the following statements:

"There are several FMS major guidelines that can be applied in the German context; these recommend various therapeutic approaches for the different treatment of FMS, including exercise (26,34,35). One of these guidelines is the EULAR guideline, which applies to the European context and recommends different non-pharmacological treatments, such as exercise, meditative movement and mind-body therapy for FMS. In this guideline, exercise is the only non-pharmacological treatment for FMS that is strongly recommended (strong for 100% agreement) based on high-quality evidence (34). The EULAR explicitly highlights aerobic and strength training, as effective types of exercise for FMS (34). In line with this, the German interdisciplinary S3 guideline on FMS emphasizes the importance of exercise (aerobic, strength and flexibility) as an intervention supported by high level-evidence and a strong recommendation (26). Moreover, the American College of Sports Medicine (ACSM) provides guidance on exercise dosing and prescription, which has been used to evaluate the adherence of exercise interventions to recommended dosage parameters in FMS (36,37). To provide a more rigorous comparison in this study, the Home Exercise (HE) program comprises all these evidence-based recommendations: aerobic, strength and stretching exercises with the recommended dosing. Both groups receive the HE program, while the intervention group additionally participates in PYM sessions, allowing evaluation of the added benefit of PYM beyond home exercises alone."

2. This study was designed as a single-blind trial, yet it also mentions that both the outcome assessors and statisticians were blinded, which is contradictory. According to the latest RCT writing guidelines, the blinding method includes four groups of people. Please explain in detail the specific subjects and procedures of the blinding method.

The scientific literature is inconsistent with regard to the meaning of the levels in the blinding procedure, but we agree that a single-blind trial cannot involve both outcome assessors and the statisticians. We have therefore removed the term "single-blind"

from the title. Instead of the terms “single-blind” or “double-blind”, we now exclusively refer to the groups of people who are blinded, in our case the outcome assessors and the statisticians (data analysts). In accordance with the SPIRIT guidelines, it is sufficient to specify who was blinded https://www.consort-spirit.org/item20a-whowasblinded.

3. Please add the assessor's qualifications and a detailed description of the assessment process.

We have added the assessors’ qualifications and expanded the assessment process description in detail in the revised manuscript with the following information:

“Baseline eligibility and ACR-criteria confirmation will be performed by ANVK, a study physician; and HSK, a physiotherapist and doctoral researcher who will conduct the primary and secondary outcome assessments at both baseline and post-intervention. Assessments will take place at baseline and at week 8 of the study, which includes the confirmation of eligibility/visit procedures, HRV measurement and completion of questionnaires. To ensure consistency across participants and visits, a structured assessment procedure will be employed, encompassing both written instructions and a checklist. For example, instructions regarding points to keep in mind prior to the measurement appointment will be provided to participants via email and telephone (e.g., avoiding alcohol, smoking, intense exercise, and heavy meals before HRV assessment). A checklist will be prepared containing instructions for the outcome assessor to follow, written in the same order and using the same wording during the measurements. Participants will primarily complete questionnaires electronically on tablet via REDCap; paper forms will be available if required and entered into REDCap by the outcome assessors. HRV measurements will be recorded under standardized conditions (quiet room, seated position, after a brief rest) and stored in REDCap. Should any artifacts requiring more than 5% correction be detected, whether due to unintended movement or speech during HRV measurement, the procedure will be repeated.”

4. This study used repeated measures ANOVA to estimate the sample size, but the effect size was calculated using “d” instead of “f” or “η2”. Please confirm this.

Thank you for noting this. The sample size calculation was based on a repeated-measures ANOVA in G*Power using Cohen’s f. We have now clarified this in the manuscript and have reported the corresponding converted value (f = 0.675) in addition to ‘d’, which was provided as it is more common and easier to interpret.

5. Please provide a detailed description of the innovativeness of this comprehensive therapy.

In our study, we developed the components of the intervention by combining various techniques (physiotherapy techniques, yoga, mindfulness) whose effectiveness in addressing the different mechanisms underlying specific FMS symptoms has been investigated in a number of studies. In terms of physiotherapy techniques, Proprioceptive Neuromuscular Facilitation (PNF), for example, can increase range of motion by reducing through pain modulation

(https://pubmed.ncbi.nlm.nih.gov/17052131/). According to the findings of a randomized controlled trial, active neurodynamic mobilization exercises can be effective in improving pain, functional status and fatigue in FMS (https://www.sciencedirect.com/science/article/pii/S0003999315004888). Nerve Gliding Exercises (NGE)may act through central hypoalgesia, improved nerve conduction, reduced perineural inflammation, and inhibition of central sensitization (https://www.sciencedirect.com/science/article/pii/S0003999315004888). Furthermore, as discussed in detail in the manuscript, numerous studies suggest that yoga and mindfulness practices may also be effective treatment options for managing FMS symptoms. Research suggests that yoga and mindfulness appear to modulate pain through various mechanisms, such as regulating attention, emotion, cognitive processes, improving ability to cope with pain, and central nervous system allostasis (https://www.sciencedirect.com/science/article/pii/S0965229925001669 , https://www.jpain.org/article/S1526-5900(17)30891-X/fulltext , https://www.sciencedirect.com/science/article/abs/pii/S0022399913003760).

Given the complex and multifactorial nature of FMS symptoms, the structure of the PYM intervention, which addresses numerous different mechanisms, and the integration of these under a single intervention framework, along with its comprehensive structure, makes it a highly innovative intervention.

We have updated the introduction section of our article by explaining how this intervention differs from others in the literature and what its innovative aspects are by adding following statements in introduction section the in revised manuscript:

"Mind-Body interventions (MBIs) such as mindfulness and yoga appear to modulate pain by altering attentional deployment and cognitive control mechanisms (17,18). They are thought improve cognitive flexibility by enabling the person to see their emotional reactions from an objective perspective without judgement, which could alleviate FMS-related symptoms (19,20). MBIs can also enhance the quality of life by improving the ability to cope with pain-related psychological problems such as stress, anxiety, and depression (21). Furthermore, allostasis has been reported as a mechanism underlying the effectiveness of MBIs in chronic pain disorders similar to FMS (16), where changes in HPA-axis activity and ANS function may lead to structural brain adaptations that facilitate coping with internal/external demands (22). Nevertheless, recent systematic reviews have found a paucity of high-quality evidence supporting MBIs, including yoga- and mindfulness-based approaches, in the management of FMS symptoms (23,24). Accordingly, more randomized controlled trials have been recommended to investigate the additive or synergistic effects of different exercise modalities or techniques in combination with MBIs (25). In addition, the German S3 guideline for FMS strongly recommends the incorporation of meditative movement therapies, including yoga, emphasizing multimodal treatment programs (26).

In line with the National Center for Complementary and Integrative Health's (NCCIH) conceptualization of integrative approaches as a coordinated multimodal care strategy (27), which integrates conventional and complementary methodologies, the current

study proposes a multimodal integrative approach. It has been developed specifically for this trial, integrating physiotherapy techniques into a structured yoga sequence and combining them with mindfulness principles. Neuromusculoskeletal function, pain-related biopsychological mechanisms, autonomic regulation, and stress-related mechanisms are targeted simultaneously. The aim is to combine physiotherapy approaches targeting pain modulation and physical function with yoga- and mindfulness-based practices that may support stress regulation, sleep, and fatigue, thereby providing an integrated approach to the multidomain symptom profile of fibromyalgia. This program, termed PhYoMind (PYM), has been developed by combining various components whose effectiveness in addressing the different mechanisms underlying specific FMS symptoms has been suggested in a number of studies. PYM integrates specific physiotherapy techniques including Proprioceptive Neuromuscular Facilitation (PNF), Nerve Gliding Exercises (NGE), Mobility Exercises (ME), and Post-Isometric Relaxation (PIR) with yoga and mindfulness. Evidence from physiotherapy research suggests that several approaches incorporated in our intervention may help prevent declines in physical function in FMS and improve symptoms such as pain, limited range of motion, balance impairments, morning stiffness and strength (28–30). For example, PNF may increase short-term range of motion via autogenic/reciprocal inhibition; while long-term mobility gains may reflect increased tolerance through pain modulation (31). NGE has been shown to improve pain, functional status and fatigue in FMS, possibly through inhibition of central sensitization, improved nerve blood flow and reduced mechanical and inflammatory stress around nerves (28). Another RCT showed that both post-isometric relaxation and myofascial release improved cervical and low back pain, mobility, and quality of life in women with FMS (32). Apart from the benefits these various techniques offer on their own or as part of physiotherapy programs, they are very well suited to yoga-based practices because they can be applied gently, gradually, and tailored to the individual's tolerance under in-person instruction and supervision. This is particularly important for FMS patients, who often have limited tolerance for high-intensity exercise (33). Given the complex and multifactorial nature of FMS symptoms, the structure of the PYM intervention, which addresses numerous different mechanisms, and the integration of these under a single intervention framework, along with its comprehensive structure, makes it a highly innovative intervention."

And in the last paragraph in introduction section, we added the following statement:

"PYM intervention stands out for its unique approach to addressing FMS symptoms; this program, in which the techniques used in physiotherapy, yoga and mindfulness components are presented as a cohesive whole rather than as discrete modules, represents a distinctive within an integrated, replicable program. Beyond this, the fact that the intervention group carries out both PYM and home exercises has the potential to yield greater synergistic benefits for the intervention group."

Minor issue
1. Please add a simple recruitment flowchart.

We have added a simple participant flow diagram in the revised manuscript (page 18).

2. The inclusion criteria should include requirements for the duration of fibromyalgia and the initial pain level.

Thank you for this suggestion. The duration of fibromyalgia was not specified in the inclusion criteria, but the duration of at least 3 months of pain duration is covered by the ACR diagnostic criteria for FMS, which we applied when screening the participants. Moreover, the participants must meet the diagnostic-level threshold for widespread pain severity required for the diagnosis, which we have verified prior to the study. Additionally, baseline pain intensity (average, worst and current pain intensity) and baseline FMS duration were collected for all participants and will be reported.

3. What is the basis for the treatment prescriptions set in the intervention group, and is there any literature or other evidence to support it?

In response to query number 5 in the 'Major issue' section, the rationale behind this intervention and all the factors considered in its planning have been explained in detail, including the basis for the treatment prescriptions.

There are a considerable number of studies that support the all components separately in the intervention for the alleviation of FMS symptoms. We hope you will find the detailed information addressing your concerns in the revised article with the following statements:

"Mind-Body interventions (MBIs) such as mindfulness and yoga appear to modulate pain by altering attentional deployment and cognitive control mechanisms (17,18). They are thought improve cognitive flexibility by enabling the person to see their emotional reactions from an objective perspective without judgement, which could alleviate FMS-related symptoms (19,20). MBIs can also enhance the quality of life by improving the ability to cope with pain-related psychological problems such as stress, anxiety, and depression (21). Furthermore, allostasis has been reported as a mechanism underlying the effectiveness of MBIs in chronic pain disorders similar to FMS (16), where changes in HPA-axis activity and ANS function may lead to structural brain adaptations that facilitate coping with internal/external demands (22). Nevertheless, recent systematic reviews have found a paucity of high-quality evidence supporting MBIs, including yoga- and mindfulness-based approaches, in the management of FMS symptoms (23,24). Accordingly, more randomized controlled trials have been recommended to investigate the additive or synergistic effects of different exercise modalities or techniques in combination with MBIs (25). In addition, the German S3 guideline for FMS strongly recommends the incorporation of meditative movement therapies, including yoga, emphasizing multimodal treatment programs (26).

In line with the National Center for Complementary and Integrative Health's (NCCIH) conceptualization of integrative approaches as a coordinated multimodal care strategy (27), which integrates conventional and complementary methodologies, the current study proposes a multimodal integrative approach. It has been developed specifically for this trial, integrating physiotherapy techniques into a structured yoga sequence and combining them with mindfulness principles. Neuromusculoskeletal function, pain-related biopsychological mechanisms, autonomic regulation, and stress-related mechanisms are targeted simultaneously. The aim is to combine physiotherapy approaches targeting pain modulation and physical function with yoga- and mindfulness-based practices that may support stress regulation, sleep, and fatigue, thereby providing an integrated approach to the multidomain symptom profile of fibromyalgia. This program, termed PhYoMind (PYM), has been developed by combining various components whose effectiveness in addressing the different mechanisms underlying specific FMS symptoms has been suggested in a number of studies. PYM integrates specific physiotherapy techniques including Proprioceptive Neuromuscular Facilitation (PNF), Nerve Gliding Exercises (NGE), Mobility Exercises (ME), and Post-Isometric Relaxation (PIR) with yoga and mindfulness. Evidence from physiotherapy research suggests that several approaches incorporated in our intervention may help prevent declines in physical function in FMS and improve symptoms such as pain, limited range of motion, balance impairments, morning stiffness and strength (28–30). For example, PNF may increase short-term range of motion via autogenic/reciprocal inhibition; while long-term mobility gains may reflect increased tolerance through pain modulation (31). NGE has been shown to improve pain, functional status and fatigue in FMS, possibly through inhibition of central sensitization, improved nerve blood flow and reduced mechanical and inflammatory stress around nerves (28). Another RCT showed that both post-isometric relaxation and myofascial release improved cervical and low back pain, mobility, and quality of life in women with FMS (32). Apart from the benefits these various techniques offer on their own or as part of physiotherapy programs, they are very well suited to yoga-based practices because they can be applied gently, gradually, and tailored to the individual's tolerance under in-person instruction and supervision. This is particularly important for FMS patients, who often have limited tolerance for high-intensity exercise (33). Given the complex and multifactorial nature of FMS symptoms, the structure of the PYM intervention, which addresses numerous different mechanisms, and the integration of these under a single intervention framework, along with its comprehensive structure, makes it a highly innovative intervention."

Additional revisions:

- We have replace the term "FM" with "FMS" in order to use the term "fibromyalgia syndrome", in order to reflect the heterogeneous causes and symptom constellation of the condition (https://register.awmf.org/de/leitlinien/detail/145-004?utm=)

- We have added a brief clarification to the Statistical analysis section regarding the exploratory analyses:
  "Given the limited sample size, no formal mediation analysis is planned as part of the confirmatory or secondary statistical analyses. Associations between fibromyalgia severity, perceived stress, and clinical outcomes may be explored descriptively or in exploratory regression models, but these analyses will be considered hypothesis-generating and interpreted with caution".

VERSION 2 - REVIEW

Reviewer 1

Name Jiang, Liming

Affiliation Shanghai University of Traditional Chinese Medicine, Shanghai Seventh People's Hospital

Date 04-Jun-2026

COI

The author has thoroughly revised the manuscript to meet the required standards; I recommend accepting this submission.

VERSION 2 - AUTHOR RESPONSE

We would like to thank Reviewer 1 for the positive assessment of our manuscript and the recommendation for acceptance. All revisions have been highlighted in the marked-up version of the manuscript, and a clean version has also been uploaded.
